# Upregulation of brain hepcidin in prion diseases: **Short title**: Hepcidin and brain iron accumulation

**DOI:** 10.1080/19336896.2021.1946377

**Published:** 2021-07-05

**Authors:** Suman Chaudhary, Ajay Ashok, Aaron S. Wise, Neil A. Rana, Dallas McDonald, Alexander E. Kritikos, Qingzhong Kong, Neena Singh

**Affiliations:** Department of Pathology, School of Medicine, Case Western Reserve University, Cleveland, Ohio, USA

**Keywords:** Hepcidin, iron, ferroportin, ferritin, brain iron

## Abstract

Accumulation of redox-active iron in human sporadic Creutzfeldt-Jakob disease (sCJD) brain tissue and scrapie-infected mouse brains has been demonstrated previously. Here, we explored whether upregulation of local hepcidin secreted within the brain is the underlying cause of iron accumulation and associated toxicity. Using scrapie-infected mouse brains, we demonstrate transcriptional upregulation of hepcidin relative to controls. As a result, ferroportin (Fpn), the downstream effector of hepcidin and the only known iron export protein was downregulated, and ferritin, an iron storage protein was upregulated, suggesting increased intracellular iron. A similar transcriptional and translational upregulation of hepcidin, and decreased expression of Fpn and an increase in ferritin expression was observed in sCJD brain tissue. Further evaluation in human neuroblastoma cells (M17) exposed to synthetic mini-hepcidin showed downregulation of Fpn, upregulation of ferritin, and an increase in reactive oxygen species (ROS), resulting in cytotoxicity in a dose-dependent manner. Similar effects were noted in primary neurons isolated from mouse brain. As in M17 cells, primary neurons accumulated ferritin and ROS, and showed toxicity at five times lower concentration of mini-hepcidin. These observations suggest that upregulation of brain hepcidin plays a significant role in iron accumulation and associated neurotoxicity in human and animal prion disorders.

## Introduction

Prion disorders are a group of neurodegenerative conditions resulting from the accumulation of PrP-scrapie (PrP^Sc^), a pathogenic isoform of the normal cellular prion protein (PrP^C^), in diseased brains. A conformational change in PrP^C^ from a mostly α-helical membrane protein to a β-sheet-rich isoform named PrP^Sc^ renders it insoluble in non-ionic detergents, and resistant to limited digestion by proteinase-K (PK). Deposits of PK-resistant PrP^Sc^ in the brain parenchyma are a hallmark of human and animal prion disorders. Prion disorders are rapidly progressive, resulting in significant neuronal death in a relatively short time. A variety of mechanisms have been proposed, some of which are only partially understood [1–4]. Among these, accumulation of redox-active iron in the brain parenchyma has been described as one of the causes of neuronal death in sporadic Creutzfeldt-Jakob disease (sCJD), a human prion disorder, and scrapie-infected animal models. It is believed that iron amplifies the neurotoxicity by catalysing the generation of highly toxic reactive oxygen species (ROS) by Fenton chemistry [3,5–14]. The underlying cause of iron accumulation, however, has remained unclear.

Several mechanisms have been proposed to explain the accumulation of iron in prion disease affected brains, including astrogliosis, microgliosis, and phagocytosis of iron-rich dead or dying neurons. Accumulated ferritin is rich in redox-active iron, creating a toxic environment for the surviving neurons [8–12]. It has remained unclear whether deposits of iron-rich ferritin are extracellular and therefore represent cellular debris, or occur within specific cells and contribute to their demise. Such a scenario would be more meaningful in developing viable therapeutic options than extracellular deposits of iron sequestered in ferritin. Moreover, an understanding of the cause of iron accumulation in neurons is likely to help in preventing such an occurrence. Recent reports suggesting local synthesis of hepcidin in the brain indicates that accumulation of iron may in fact be initiated within neurons [15–19], a possibility that requires further exploration.

Hepcidin is mainly a hepatic peptide hormone that maintains iron levels within a narrow range in the peripheral circulation by regulating the expression of ferroportin (Fpn), the only known iron export protein. The increase in iron saturation of serum transferrin (Tf-iron), the principal iron carrier protein, upregulates hepcidin, downregulating Fpn by binding and inducing its internalization and degradation. This limits uptake of additional iron from intestinal epithelial cells, and blocks release of stored iron from macrophages and other storage cells. The opposite scenario takes effect when Tf-iron falls below a certain range [20,21]. The brain is protected from fluctuations in serum iron by the blood–brain barrier (BBB) and blood-cerebrospinal fluid (CSF) barriers, allowing regulated exchange of iron through iron uptake and export proteins. These proteins respond to iron saturation of CSF Tf, thus protecting the neurons from the toxic effects of excess iron and iron-catalysed ROS. Local synthesis of hepcidin by astrocytes and other brain cells suggests additional regulation of iron locally within the brain. Expression of Fpn on the neuronal plasma membrane suggests regulation of neuronal iron by local hepcidin through its paracrine action [15–19].

However, hepcidin is also upregulated by cytokines, especially IL-6, IL-1β, and TGFβ1 & β2 [22–25], and the signal from cytokines supersedes that of Tf-iron. This is the principal cause of anaemia of chronic inflammation where cytokine-mediated upregulation of hepcidin limits uptake of additional iron and release from iron stores despite functional iron deficiency. Since sCJD and mouse scrapie are invariably associated with neuroinflammation [26–31], it is likely that cytokine-mediated upregulation of local hepcidin contributes to the accumulation of iron and upregulation of ferritin in diseased brains. Here, we explored this possibility using a combination of brain tissue from sCJD cases and scrapie-infected mouse models, human neuroblastoma cells, and primary neurons cultured from mouse brains.

## Results

### Scrapie-infected mice show upregulation of brain hepcidin

**Figure 1.**
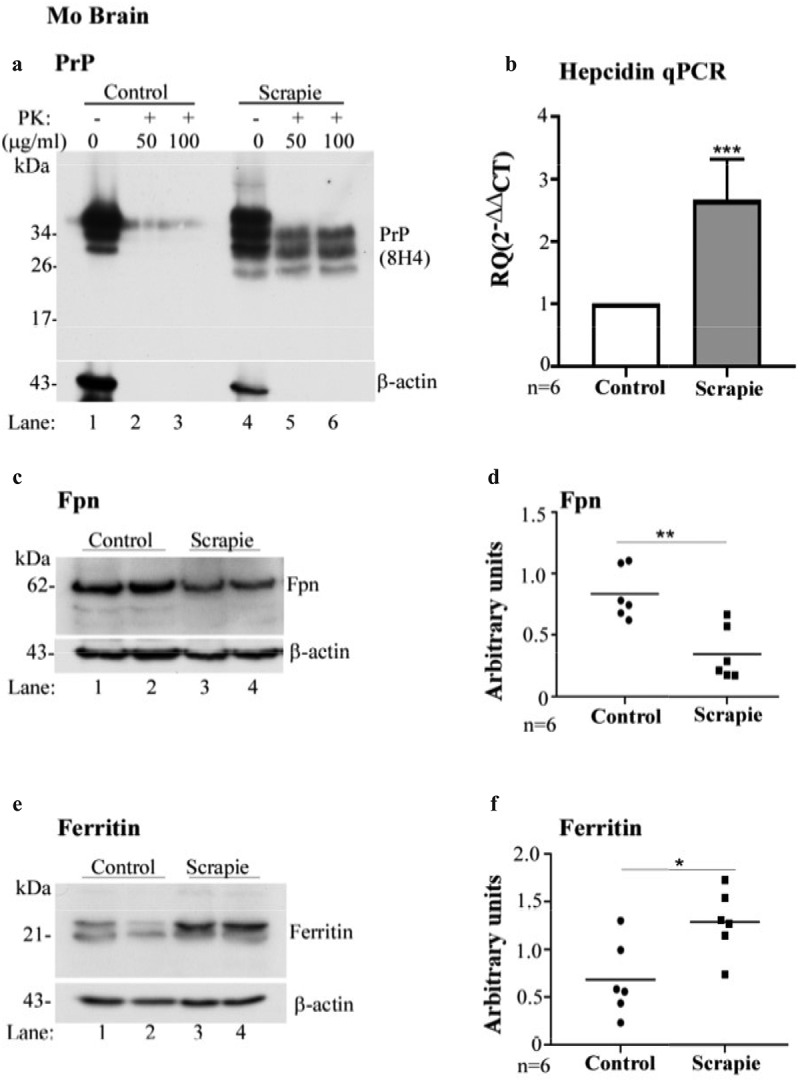
**Hepcidin is upregulated in scrapie infected mouse brains**. (a) Control and scrapie infected mouse brains harvested at end-stage disease were treated with proteinase K (50 and 100 μg/ml) and analysed using WB. Probing for PrP (8H4) reveals complete digestion of control brain tissues with PK (lanes 1–3), while faster migrating PK-resistant bands are detected in PrP^Sc^-infected tissue (lanes 4–6). (b) Amplification of hepcidin by RT-qPCR from scrapie infected mouse brains shows 2.6-fold upregulation relative to controls. GAPDH was used as a house-keeping gene. (c) Western blotting of mouse brain homogenates and probing for Fpn shows decreased expression in scrapie-infected samples relative to controls (lanes 3, 4 vs. 1, 2). (d) Densitometry after normalization with β-actin shows a significant decrease in Fpn expression in diseased brains relative to controls. (e) Western blotting and probing of the same samples for ferritin shows increased expression in scrapie-infected samples relative to controls (lane 3, 4 vs. 1, 2). (f) Densitometry after normalization with β-actin indicates significant upregulation of ferritin in scrapie-infected brain tissues relative to controls. A representative blot is shown in A, C, and E. Values are mean ± SEM of the indicated n. *p < 0.05, **p < 0.01, ***p < 0.001

Imbalance of iron homoeostasis in scrapie-infected mouse and hamster models has been reported previously [14,32]. To evaluate whether increase in hepcidin is the underlying cause, wild-type FVB/NJ mice were inoculated with scrapie-infected brain homogenate intracerebrally. Control mice received the same amount of normal brain homogenate. At end-stage disease, the brains were harvested, and homogenates treated with 50 and 100 μg/ml of proteinase-K (PK) for 1 h. The reaction was terminated with protease-inhibitors, and digested lysates were fractionated on SDS-PAGE and analysed by Western blotting (WB). Probing with PrP antibody, 8H4 indicated PK-resistant, faster migrating bands of PrP^Sc^ in infected samples as expected. PrP^C^ from normal brain homogenates was digested completely (Figure 1 A, lanes 4–6 vs. 1–3).

To evaluate any change in hepcidin at the transcriptional level, total mRNA was extracted from normal and PrP^Sc^-infected animals, and subjected to real-time quantitative polymerase-chain reaction (RT-qPCR) with mouse hepcidin-specific primers. Quantification revealed 2.6-fold upregulation of hepcidin in scrapie-infected samples relative to controls, suggesting increased synthesis of local hepcidin in diseased brain tissue (Figure 1 B).

The physiological significance of these observations was determined by evaluating the expression of Fpn in control and scrapie-infected brain homogenates by Western blotting. Control and PrP^Sc^-infected whole brain homogenates prepared in PBS were depleted of red blood cells (RBC) with hypotonic saline, and washed with PBS to eliminate contaminating serum proteins before homogenizing with lysis buffer. Equal amount of protein from control and PrP^Sc^-infected lysates was fractionated by SDS-PAGE, and analysed by Western blotting. Probing for Fpn showed a specific band migrating at the expected molecular mass (Figure 1 C, lanes 3 & 4 vs. 1 & 2). Quantification of band intensity by densitometry after normalization with β-actin showed significant downregulation in scrapie infected samples relative to controls (Figure 1 D). A new set of samples was evaluated similarly for ferritin expression, and significant upregulation was noted in PrP^Sc^-samples relative to controls, probably due to an increase in the iron content due to decreased export through Fpn (Figure 1 E, lanes 3 & 4 vs. 1 & 2; Figure 1 F).

### Hepcidin in upregulated in sCJD brain tissue

**Figure 2.**
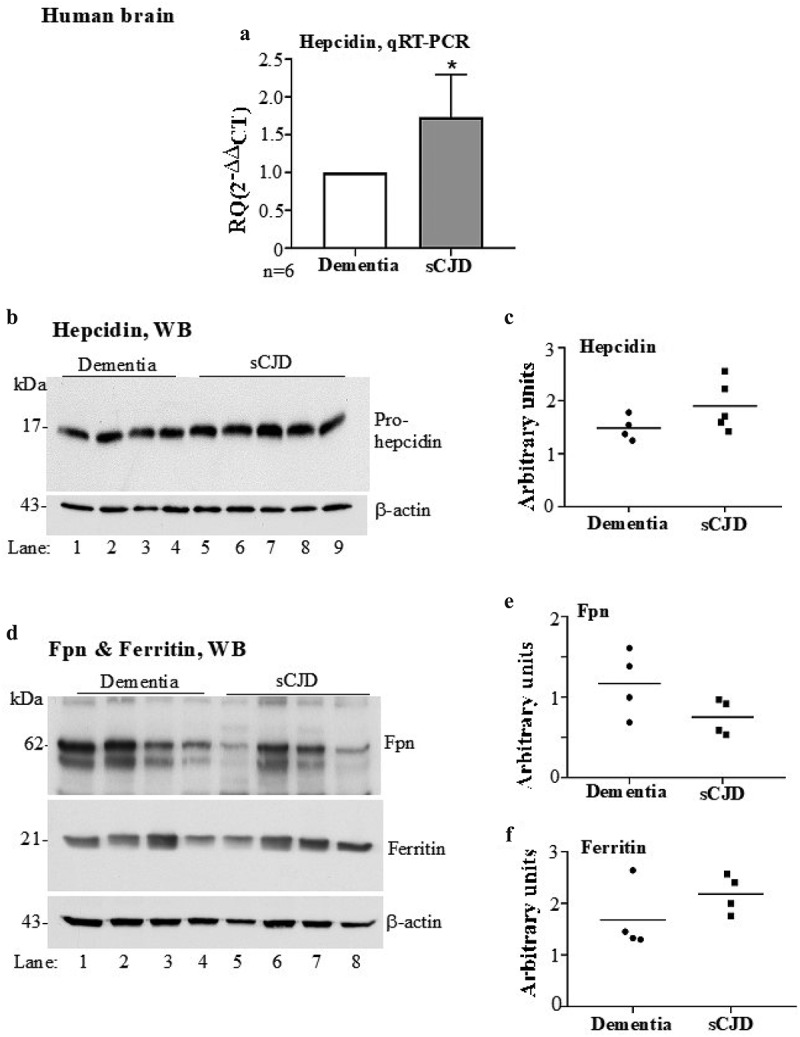
**Hepcidin is upregulated in autopsy brains from sCJD cases**. (a) Amplification of hepcidin by RT-qPCR shows 1.7-fold upregulation relative to controls. GAPDH was used as a house-keeping gene. (b) Western blotting of brain lysates from sCJD and dementia samples and probing for hepcidin showed an increase in pro-hepcidin expression in sCJD samples relative to dementia controls (lanes 5–9 vs. 1–4). (c) Densitometry after normalization with β-actin revealed an upward trend in sCJD samples. (d) Western blotting of fresh samples from the same lysates and probing for Fpn showed decreased expression in sCJD samples relative to dementia controls (lanes 5–8 vs. 1–4). Re-probing of the same membrane for ferritin showed increased expression in sCJD samples relative to dementia controls (lanes 5–8 vs. 1–4). (E & F) Quantification of band intensity after normalization with β-actin showed a decrease in Fpn, and an increase in ferritin expression. Values are mean ± SEM of the indicated n. *p<0.05

To evaluate whether a similar increase in hepcidin contributes to the accumulation of brain iron in sCJD [14], autopsy brain tissue of confirmed sCJD cases and dementia controls was clarified of RBCs and serum proteins as above. Equal amounts of total mRNA extracted from control and sCJD brains was subjected to RT-qPCR with primers specific for human hepcidin. Surprisingly, a 1.7-fold upregulation of hepcidin was observed in sCJD tissue relative to dementia controls (Figure 2 A)

Subsequently, equal amounts of protein from clarified brain homogenates prepared in the lysis buffer were analysed for protein expression by Western blotting. Probing with antibody specific for human hepcidin revealed a discrete band of pro-hepcidin migrating at ~17 kDa (Figure 2 B, lanes 5–9 vs. 1–4) [33,34]. Quantification by densitometry after normalization with β-actin showed an increase in hepcidin expression in sCJD samples relative to dementia controls (Figure 2 C).

To evaluate whether the increase in hepcidin has an effect on the expression of Fpn and ferritin, brain lysates from sCJD and dementia controls were fractionated as above, and probed for Fpn and ferritin. Even though statistically non-significant, it is notable that Fpn showed a downward trend, and ferritin an upward trend in sCJD samples relative to dementia controls (Figure 2 D, lanes 5–8 vs. 1–4). These observations were confirmed by quantification of band intensity after normalization with β-actin (Figure 2 E & 2 F).

### Hepcidin induces iron-mediated toxicity in neuronal cells

**Figure 3.**
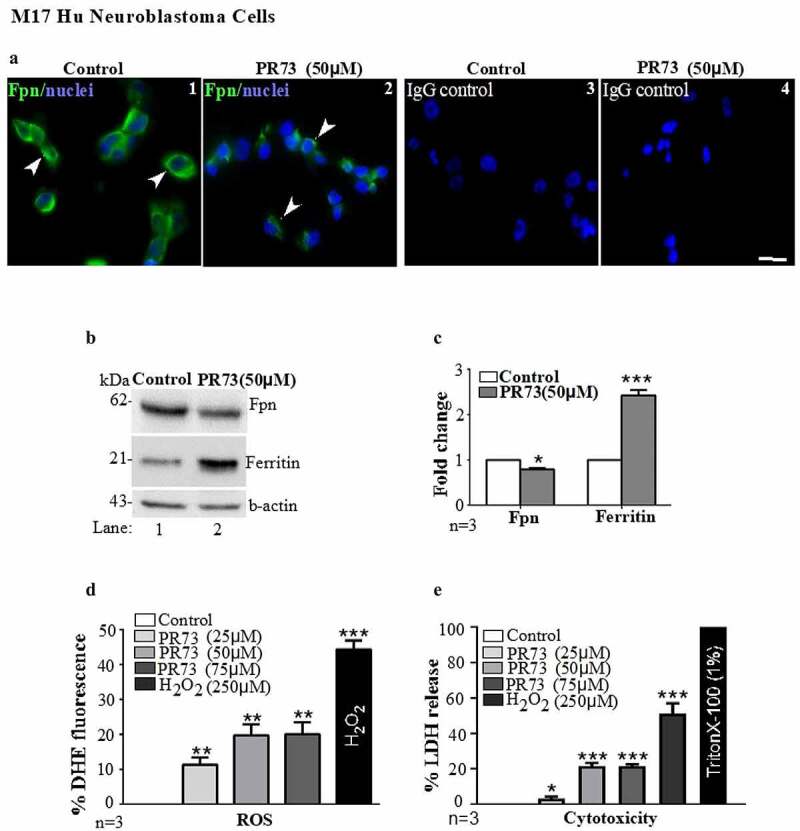
**Mini-hepcidin increases iron-mediated ROS and is toxic to M17 cells**: (a) M17 cells cultured on cover-slips were exposed to vehicle or 50 µm PR73 for 16 h, and immunostained for Fpn. Vehicle-exposed cells show normal distribution of Fpn on the plasma membrane (panel 1), and internalization and degradation by PR73 (panel 2). Control cells immunoreacted with isotype-specific antibody show no reaction (panels 3 & 4). Scale bar: 25 µm. (b) Western blotting of M17 lysates exposed to vehicle of PR73 as above show downregulation of Fpn by PR73 (lane 2 vs. 1). Re-probing of the same membrane for ferritin shows upregulation in cells exposed to PR73 (lane 2 vs. 1). A representative blot is shown. (c) Densitometry after normalization with β-actin confirms significant down regulation of Fpn and upregulation of ferritin relative to controls. Values are mean ± SEM of the indicated n. *p < 0.05, ***p < 0.001. (d) Increase in intracellular ROS in M17 cells exposed to increasing concentrations of PR73 was quantified by estimating the change in fluorescence intensity of DHE-loaded cells relative to cells exposed vehicle (0%) or 250 µM of H_2_O_2_ (44%) in complete medium. Exposure to 25 µM PR73 increased the fluorescent intensity to 11%, with a further increase to 20% by 50 and 75 µM PR73, and 44% with 250 µM H_2_O_2_. (e) Release of LDH in the medium as a measure of cytotoxicity revealed 3% with 25 µm PR73, increasing to 21% with 50 and 75 µm PR73 relative to vehicle exposed cells at 0%, and Triton X-100 exposed cells at 100%. Values represent three independent estimations of triplicate cultures. *p < 0.05, **p < 0.01, ***p < 0.001

To understand the role of hepcidin in brain iron accumulation, human M17 neuroblastoma cells and primary mouse brain cultures enriched for neurons were exposed to a synthetic analogue of hepcidin (PR73) with a longer half-life than serum hepcidin [35]. Thus, sub-confluent cultures of M17 cells plated on cover-slips were exposed to vehicle or 50 µM of PR73 for 16 h, and expression of Fpn was evaluated by immunostaining-fixed cells with an Fpn-specific antibody followed by a FITC-conjugated secondary antibody. Cells exposed to the vehicle showed plasma membrane expression of Fpn as expected. Exposure to PR73, on the other hand, resulted in internalization and subsequent degradation of Fpn (Figure 3 A, panels 1 & 2). No reaction was observed in cells stained in parallel with isotype-specific primary antibody followed by FITC-conjugated secondary antibodies (Figure 3 A, panels 3 & 4).

Subsequently, lysates prepared from M17 cells exposed to the vehicle or 50 µM of PR73 for 16 h were processed for Western blotting, and probed for Fpn and ferritin. As noted in immunostained cells, Fpn levels were lower in cells treated with PR73 relative to vehicle-treated cells (Figure 3 B, lane 2 vs. 1). The re-probing of the same membrane for ferritin, on the other hand, showed upregulation by PR73 relative to vehicle treated controls (Figure 3 B, lane 2 vs 1). Quantification of the band intensity after normalization with β-actin showed significant downregulation of Fpn and upregulation of ferritin by PR73 (Figure 3 C). Finally, M17 cells were cultured in 96-well plates, and triplicate cultures were treated with increasing concentrations of PR73 (25 µM, 50 µM and 75 µM) for 16 h. Negative controls received the vehicle dissolved in medium, and positive controls were exposed to 250 µM of H_2_O_2_. At the indicated time, cells were washed, and loaded with dihydroethidium (DHE), a ROS-sensitive dye for 40 minutes [36,37]. After a final wash with PBS, the change in fluorescence of DHE by the superoxide anion released by Fenton chemistry was measured in a plate reader. Fluorescence intensity of DHE increased by 11% with 25 µM of PR73, and doubled to 20% with 50 µM and 75 µM of PR73 as compared to 0% in vehicle treated cells and 44% in cells exposed to H_2_O_2_ (Figure 3 D)

A parallel set of cells was treated with PR73 or vehicle as above, and cytotoxicity by PR73 was measured by the lactate dehydrogenase (LDH) assay. Triton X-100 was added to triplicate cultures as a positive control. The plate was centrifuged, and the medium collected to estimate the release of LDH. Quantification revealed 3% cell death with 25 µM of PR73, and an increase to 21% with 50 µM and 75 µM of PR73 relative to 0% in vehicle-treated control cells, and 100% in cells exposed to 1% Triton-X-100 (Figure 3 E).

The above results suggest that PR73 increases ROS, which plateaus at ~50 µM. A similar pattern in the cytotoxicity assay indicates ROS-mediated death in these cells, indicating that downregulation of Fpn by hepcidin increases intracellular iron, iron-mediated ROS, and cytotoxicity.

**Figure 4.**
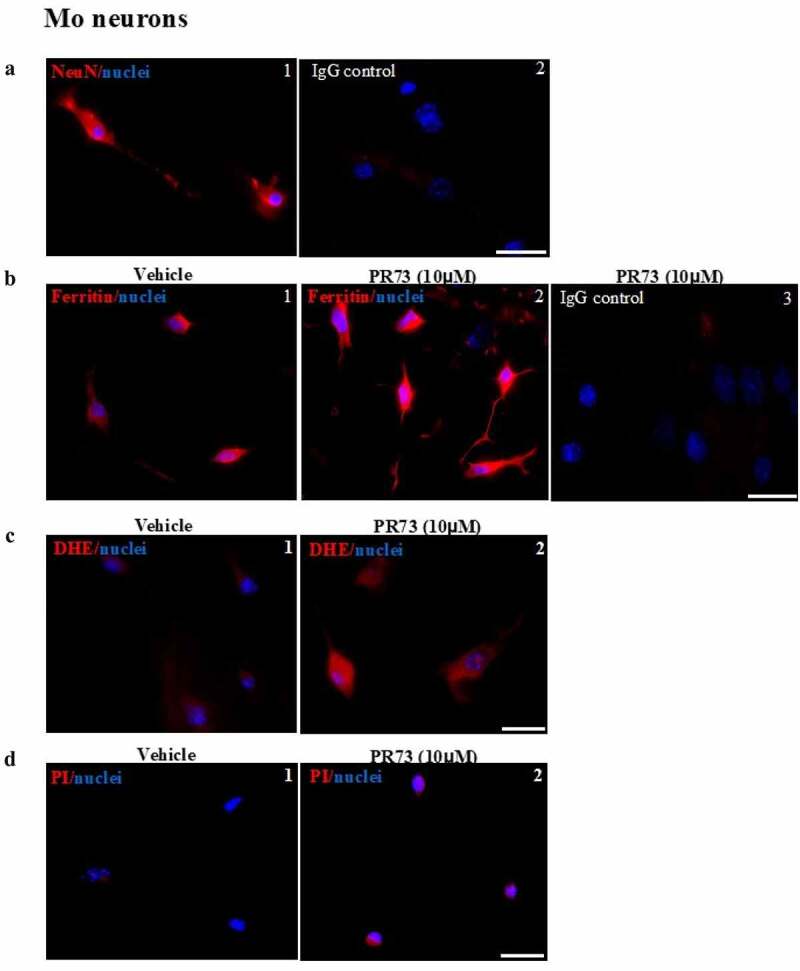
**Mini-hepcidin increases iron-mediated ROS and is toxic to primary mouse neurons**. (a) Mouse brain primary neurons cultured on cover-slips were reacted with NeuN, a neuron specific antibody. A clear reaction is detected in neuronal cell bodies and axons (panel 1). No reaction is detected with isotype specific antibody (panel 2). Scale bar: 25 µm. (b) Exposure of neuronal cultures to 10 µM of PR73 for 4 h results in strong reactivity for ferritin relative to vehicle treated cells (panel 2 vs. 1). No reaction is detected in cells immunostained with isotype-specific antibody (panel 3). (c) Primary neuronal cells exposed to vehicle or 10 µM of PR73 for 4 h followed by DHE show intense fluorescence in PR73 exposed cells, indicating generation of ROS (panel 2 vs. 1). (d) Nuclei of cells showing a positive reaction for ROS react with propidium iodide, indicating cytotoxicity. Scale bar: 25 µm

These results were confirmed in primary neuronal cells isolated from the brains of new-born mouse pups. Mixed cultures were enriched for neurons, and cells cultured on coverslips were exposed to vehicle or 10 µM PR73 for 4 h and immunostained for neuronal nuclear protein (NeuN) and ferritin. Several cells showed a positive reaction for NeuN, confirming their neuronal origin. Cells reacted in parallel with isotype-specific antibody did not show any reaction (Figure 4 A, panels 1 & 2). Immunostaining for ferritin showed intracellular reaction, which increased significantly in PR73 treated cells. Cells exposed to isotype-specific antibody did not show any reaction (Figure 4 B, panels 1–3). An independent set of cells exposed to vehicle or 10 µM PR73 for 4 h were loaded with DHE as above, and the change in fluorescence intensity was measured. As in M17 cells, exposure to PR73 increased intracellular fluorescence significantly, indicating generation of ROS (Figure 4 C, panels 1 & 2). Several neurons in the PR73 treated culture showed positive staining for propidium iodide, indicating toxicity by this treatment (Figure 4 D, panels 1 & 2).

## Discussion

Our observations demonstrate transcriptional upregulation of hepcidin in sCJD brain tissue and PrP^Sc^-infected mouse brains. The consequent downregulation of Fpn resulted in accumulation of iron upregulation of cytosolic ferritin. These observations were replicated in human M17 cells and primary cultures of mouse neurons which responded to mini-hepcidin by downregulating Fpn and increasing ferritin expression. Hepcidin-mediated accumulation of iron increased ROS and induced cytotoxicity in M17 cells and primary mouse neurons in a dose-dependent manner, suggesting a similar sequence of events in diseased brains. These observations suggest local hepcidin as a contributing factor in brain iron accumulation and neurotoxicity in human and animal prion disorders.

Upregulation of iron-rich ferritin in sCJD and PrP^Sc^-infected animal models has been known for some time [3,8–11,14]. In animal models, accumulation of iron-rich ferritin increased with disease progression [14]32]. In sCJD brains, aggregated ferritin co-purified with PrP^Sc^, and resisted limited digestion with PK and digestive enzymes. Aggregates of ferritin and PrP^Sc^ were internalized by intestinal epithelial cells together, suggesting that ferritin acts as a carrier for PrP^Sc^ [38]. Surprisingly, chelation of iron from sCJD brain homogenates increased the susceptibility of PrP^Sc^-ferritin aggregates to PK, implicating iron in imparting PK-resistance in addition to toxicity by ROS [14,39]. Aggregation of ferritin was also noted in retinal lysates from Chronic Wasting disease (CWD)-infected mouse modes, a prion disorder of the deer and elk population. Here, upregulation of ferritin followed microglial activation, indicating a role of cytokines in this process [40]. The accompanying upregulation of transferrin (Tf), an iron uptake protein in sCJD brain homogenates and CWD-infected retinal lysates suggested sequestration of iron in a biologically unavailable form, creating functional iron deficiency [14,40]. The consequent uptake of additional iron from the peripheral circulation is expected to exacerbate iron accumulation, creating an ongoing cycle [41]. In cell models infected with PrP^Sc^, aggregates of ferritin were detected in lysosomal structures, where it was presumed to co-aggregate with PrP^Sc^ and acquire PK-resistance [14]. Other studies reported increase in iron and oxidative stress [11], and upregulation of ferritin in PrP^Sc^-infected mice. However, none of the above studies were able to identify whether accumulated iron was from cellular debris, or increase in intracellular iron.

Our data demonstrating transcriptional upregulation of hepcidin in PrP^Sc^-infected mouse models and sCJD brain homogenates suggests intracellular accumulation of iron in cells that express Fpn, including neurons. Local synthesis of hepcidin by astrocytes has been known for some time [16,18,19] though the implications of this observation on brain iron metabolism have remained elusive. It is plausible that increased synthesis of hepcidin by astrocytes downregulates Fpn on neuronal plasma membrane by its paracrine activity, resulting in accumulation of iron and upregulation of ferritin. Downregulation of Fpn and upregulation of ferritin in diseased brain homogenates, and our observations on M17 neuroblastoma cells and primary mouse neurons exposed to mini-hepcidin support this assumption. Leakage of peripheral hepcidin from the BBB at end-stage disease is expected to amplify the effect of brain hepcidin, creating further accumulation of iron in neurons. Hepcidin-mediated increase in iron generates ROS and is cytotoxic, partly explaining neuronal death in diseased brains.

A clear difference in the upregulation of hepcidin in mouse brain homogenates relative to sCJD samples is mainly due to the lack of post-mortem interval in mice. Leakage across the blood-brain-barrier is expected in both cases though it would be limited in mice because of immediate processing of mouse brain homogenates. It is unlikely that any difference in liver hepcidin or other stimuli is responsible for the upregulation of hepcidin play a role.

What might be the cause of upregulation of hepcidin in prion disorders? The most likely possibility is neuroinflammation and cytokine release associated with sCJD and mouse scrapie [25,26,31,42,43]. It is notable that the cytokines IL-6, IL-1β, and TGFβ1 & β2 are transcriptional activators of hepcidin by the JAK/STAT and SMAD pathways [44,45], and the signal from cytokines supersedes the signal from Tf-iron [46–48], resulting in upregulation of hepcidin despite restricted availability of iron as in the anaemia of chronic inflammation. Thus, cytokine-mediated upregulation of hepcidin is likely to downregulate Fpn on neuronal cells [49], increasing ROS and associated toxicity.

In conclusion, our observations suggest that brain hepcidin contributes to neuronal iron accumulation and ROS-mediated toxicity in prion disease affected brains. Additional studies are necessary to define the cell types that upregulate hepcidin, and the triggers for hepcidin upregulation. This information will help in identifying strategies aimed at lowering hepcidin or Fpn stabilizing agents to reduce iron accumulation in diseased brains. Several such agents are currently undergoing clinical trials for systemic disorders of iron overload, which could be modified for use in the brain.

## Methods

### Ethics statement

Human brain tissue from dementia cases was obtained from NIH-sponsored NeuroBioBank at the University of Maryland and Mount Sinai NBTR Tissue distribution centre. Tissue from sCJD cases and dementia controls was obtained from the Centers for Disease Control and Prevention (CDC)-approved National Prion Disease Pathology Surveillance Center (NPDPSC) at Case Western Reserve University (CWRU). Informed consent was obtained by NPDPSC for harvesting brains at autopsy. All experiments were performed in compliance with the tenets of the Declaration of Helsinki. Wild-type C57BL/6 J and FVB/NJ mice were kept in Association for Assessment and Accreditation of Laboratory Animal Care (AAALAC)-approved Animal Resource Center at CWRU School of Medicine under a 12 h day-night cycle. Specific animal procedures were approved in the animal protocol number 2015–0027. The mice were provided ad libitum access to water and food. All experiments were approved by the Institutional Animal Care and Use Committee in accordance with provisions of the Animal Welfare Act and Guide for the Care and Use and of Laboratory Animals, and the US Government Principles for the Utilization and Care of Vertebrate Animals used in Testing, Research and Training.

### Sample details

Frozen post-mortem brain tissue from dementia and sCJD cases of similar age range was analysed. Details are provided in Table 1.

**Table 1.** Dementia and sCJD brain tissue

Serial #	Sex	Age (y)	PMI (h)	Diagnosis
1456	F	85	6	Dementia
5714	F	94	33	Hippocampal sclerosis dementia
6161	M	70	20	Dementia
6162	F	82	5	Dementia; Seizure Disorder
6176	F	68	11	Dementia, Unspecified
6204	M	77	11	Dementia, Unspecified
6213	M	76	19	Dementia, Unspecified
6222	M	88	6	Dementia, Unspecified
6254	F	88	20	Dementia, Unspecified
6275	F	85	27	Dementia, Unspecified
6316	F	77	5	Dementia-Frontotemporal
917	F	70	7	Dementia, Unspecified
98,901	M	70	5	Dementia, Alcohol-induced
3762	M	75	73	Dementia, Alcohol-induced
63,578	M	79	48	Dementia, Senile, delusional
3983	M	67	NA	sCJDVV1
0819	F	75	5	sCJDVV1-2, stroke
0977	F	73	15	sCJDMV2, Parkinson’s Disease
1038	M	59	9	sCJDMV1-2
1420	M	59	4	sCJDMM1, Rapid Dementia

### Antibodies and chemicals

HRP-conjugated secondary antibodies (anti-mouse (NA931V) & anti-rabbit (NA934V)) were from GE Healthcare, hydrogen peroxide (H_2_O_2_) (H1009), proteinase K (P2308), and phenylmethanesulfonyl fluoride (PMSF) (P7626-5 G) were from Sigma Aldrich, USA. Triton X-100 (BP151-500) was from Fisher scientific, USA. Mini-hepcidin PR73 was a kind gift of Elizabeth Nemeth and Thomas Ganz (UCLA). Details of all antibodies used in the study are provided in Table 2.

**Table 2.** List of antibodies used in the study

Antibody	Host species*	Species reactivity*	Company	Cat. #	Dilution**
PrP (8H4) IgG2b	m	m, h, b, r, s	Sigma Aldrich, USA	P0110	WB-1/250
Ferritin	Rb	h	Sigma Aldrich, USA	F5012	WB-1/1000 ICC-1/100
Ferroportin	Rb	h, m, r, b	Novus biologicals, USA	NBP1-21,502	WB-2/1000
Hepcidin	Rb	h, m	Novus biologicals, USA	NBP1-59,337	WB-7/1000
NeuN	Rb	h, m, r	Novus biologicals, USA	NBP1-77686SS	ICC-1/100
β-actin	m	All species	Millipore, USA	MAB1501	WB-1/5000
Alexa Fluor 546	g	Rb	Invitrogen, USA	A11071	ICC- 1/1000
Alexa Fluor 488	g	Rb	Invitrogen, USA	A11070	ICC- 1/1000
Isotype Control	Rb	No known specificity	Abcam, USA	ab37415	ICC- 1/100

* m: mouse, Rb- rabbit, h: human, r: rat, b: bovine, s: sheep, g: goat ** WB-western blotting, ICC-immunocytochemistry

### Infection with scrapie

Wild-type female FVB/NJ mice were injected intracerebrally with 30 µl of 1% brain homogenate from mice infected with the 139A strain of scrapie or normal brain homogenate as described [14,32]. The mice were monitored daily and sacrificed when signs of disease appeared. When clear signs of prion disease appeared, the mice were euthanized, the brain tissues were taken, frozen immediately on dry ice, and stored at −80°C before use. Aged matched untreated wild-type female FVB/NJ mice were used as the controls.

### RT-qPCR

Total RNA was isolated from 20 mg of mouse brain tissue and 50 mg of post-mortem non-dementia and sCJD brain tissues, using TRIzol® extraction (15,596,026, ThermoFisher Scientific, USA) protocol, as described previously [50]. The concentration and quality of extracted RNA was determined using Nano-drop 2000 c (ThermoFisher Scientific, USA). Traces of genomic DNA were removed by treating the RNA with DNAse. RNA was then converted to cDNA using SuperScript® IV Reverse Transcriptase Kit (18,090,200, Invitrogen, USA). RT-qPCR was performed on a Biorad C1000 Touch Thermal Cycler (CFX96^TM^ Real-Time System). PCR reactions were prepared in triplicated using 2 µL cDNA withBrightGreen 2X QPCR MasterMix (MasterMix-R) obtained from Applied Biological Materials Inc. (abm), USA and 10 µM of gene-specific primers in a total volume of 20 mL. Primers: Ms *HAMP*-F: GCACCACCTATCTCCATCAACAGA-3ʹ, Ms *HAMP*-R: GGTCAGGATGTGGCTCTAGGCTAT-3ʹ. Ms Gapdh-F: AACTTTGGCATTGTGGAAGG-3ʹ, Ms Gapdh-R: ACACATTGGGGGTAGGAACA-3ʹ, hu *HAMP*-F: 5ʹ-CCTGACCAGTGGCTCTGTTT-3ʹ, hu *HAMP*-R: 5ʹ-CACATCCCACACTTTGATCG-3ʹ, hu Gapdh-F: 5ʹ-GAGTCAACGGATTTGGTCGT-3ʹ, hu Gapdh-R: 5ʹ-GGTGCCATGGAATTTGCCAT-3ʹ were obtained from Integrated DNA technologies, USA. 40 cycles of cycling programme comprising enzyme activation at 95°C for 10 min, denaturation at 95°C for 15 secs followed by annealing/extension at 60°C for 60 sec were conducted. Relative gene expression normalized to GAPDH was calculated using an excel sheet. The results were assessed and graphically presented using GraphPad Prism (Version 5.0) software (GraphPad Software Inc., USA).

### Cell culture

Human neuroblastoma cell line BE(2)-M17 (M17) was obtained from ATCC (CRL-2267^TM^) and cultured in Minimum Essential Medium (12,571,063, Thermofisher, USA) supplemented with 10% foetal bovine serum (10,082,147, Invitrogen-Gibco, USA) and 1% penicillin/streptomycin (15,140,122, Invitrogen-Gibco, USA). Mouse primary neurons were isolated and cultured as previously described [51,52]. In brief, C57BL/6 J neonatal pups were rinsed with 70% ethanol and decapitated using sterile scissors. Subsequently, whole brain was scooped out and meninges were removed. The brain tissue was dissociated by trituration and centrifuged at low speed. The cell pellet was cultured in neurobasal medium containing B27 supplement (21,103–049, 17,504–044), 0.5 mM Glutamine solution (25,030–149), Penicillin/Streptomycin (Invitrogen-Gibco, USA), and 10% Heat Inactivated Donor Horse Serum (S12150H, Atlanta Biologicals, USA) on poly-L-Lysine coated coverslips. Neuronal cells were enriched by gradually reducing the serum concentration and culturing under serum-free conditions.

### Western blotting

Western blotting was performed essentially as described [53–55]. For control and scrapie infected mouse and human dementia and sCJD brain tissues, RBCs were lysed with hypotonic saline, and residual haemoglobin and serum proteins washed with PBS. Brain tissue was either processed for mRNA isolation, or homogenized in RIPA buffer (50 mM Tris-HCl pH 7.4, 150 mM NaCl, 1% NP-40, 0.5% sodium deoxycholate, 0.1% SDS) for Western blot analysis. For PK-digestion, brain homogenates without protease inhibitors were incubated with 50 or 100 mg/ml of PK at 37°C for 1 h. Digestion was stopped with the addition of 1 mM PMSF and protease inhibitor cocktail. Untreated and PK-digested samples were fractionated by SDS-PAGE followed by Western blotting. PrP bands were detected by probing with 8H4. Likewise, cell lysates from control (vehicle treated) and 50 µM PR73 treated human BE(2)-M17 cells (from ATCC) were processed for Western blotting and probed for ferritin and Fpn. The bands of interest were visualized with HRP-conjugated secondary antibody and chemiluminescent substrate (34580, Thermofisher, USA). A fixed exposure time was maintained for control and scrapie infected mouse brains and for vehicle and PR73 treated M17 cell lysates. Quantification of protein bands was performed by densitometry after normalization with β-actin using the UN-SCAN-IT gels (version6.1) software and ImageJ Software, and presented graphically using GraphPad Prism (Version 5.0) software (GraphPad Software Inc., USA) or Microsoft Excel.

### Immunocytochemistry

Immunocytochemistry was performed as described previously [53]. Briefly, M17 cells were exposed to vehicle or PR73 (50 µM) for 16 h, and primary mouse neurons to 10 µM of PR73 for 4 h and fixed in 4% paraformaldehyde. M17 cells were immunoreacted with antibody specific for Fpn. Neuronal cells were immunostained with the neuron-specific antibody NeuN and anti-ferritin antibody followed by the species-specific Alexa Fluor-conjugated secondary antibody. The nuclei were stained with Hoechst (33,342, Invitrogen, USA) or propidium iodide. Stained samples were mounted in Fluoromount-G (Southern Biotech, USA) and imaged with Leica inverted microscope (DMi8). Each experiment was repeated 3 times, and a representative image from five different fields is shown. Control samples were reacted with isotype-specific primary antibody and the same secondary antibody.

### Lactate Dehydrogenase (LDH) Assay

LDH assay was performed with the commercially available LDH detection kit (Roche-11644793001). M17 cells were treated with 25, 50 and 75 µM of PR73 for 16 h in a 96 well plate. Control cultures received vehicle, H_2_O_2_ (250 µM), or Triton X-100. Subsequently, the microplate was centrifuged at 1000 rpm for 10 min to obtain cell-free supernatants, which were incubated for 30 min with LDH substrate in the dark. Release of LDH was quantified at 492 nm with reference wavelength of 620 nm using BioTek Synergy 4 microplate reader.

### Estimation of ROS

Release of superoxide in response to PR73 treatment was analysed using the fluorescent probe dihydroethidium (DHE; 309,800, Sigma Aldrich, USA) as described [36,37]. In short, M17 cells or primary neurons were treated with different concentrations of PR73, H_2_O_2_, or 1% Triton-X 100, and loaded with 20 μM DHE in Krebs-HEPES buffer at 37°C with 5% CO_2_ for 40 min. Cells were washed with PBS, and production of ROS was detected by quantifying the change in fluorescence of DHE at 592 nm in a microplate reader (Synergy 4 (Biotek), USA).

Primary Neuronal cells incubated with DHE were fixed in 4% paraformaldehyde and stained with Hoechst (#33,342, Invitrogen, USA), Excitation/emission for DHE is 535/610 nm. Staining with propidium Iodide (PI) (P3566, Thermofisher scientific) was performed as described [56].

## List of Abbreviations

BBB: Blood-Brain Barrier, DHE: Dihydroethidium, Fpn: Ferroportin, IL: Interleukin, LDH: Lactate Dehydrogenase, NeuN: Neuronal Nuclear Protein, PK: Proteinase K, PrP^C^: Cellular Prion Protein, PrP^Sc^: Prion Protein Scrapie, ROS: Reactive Oxygen Species, sCJD: Sporadic Creutzfeldt-Jakob Disease, Tf: Transferrin, TGFβ: Transforming growth factor-beta.
